# Characterization of human periodontal ligament cells cultured on three-dimensional biphasic calcium phosphate scaffolds in the presence and absence of L-ascorbic acid, dexamethasone and β-glycerophosphate *in vitro*

**DOI:** 10.3892/etm.2015.2706

**Published:** 2015-08-24

**Authors:** SHAOFENG AN, YAN GAO, JUNQI LING

**Affiliations:** Department of Operative Dentistry and Endodontics, Guanghua School of Stomatology, Hospital of Stomatology, Sun Yat-sen University; Guangdong Provincial Key Laboratory of Stomatology, Guangzhou, Guangdong 510055, P.R. China

**Keywords:** human periodontal ligament cells, osteoblastic differentiation, biphasic calcium phosphates, scaffolds, biocompatibility

## Abstract

The aim of this study was to evaluate the effect of porous biphasic calcium phosphate (BCP) scaffolds on the proliferation and osteoblastic differentiation of human periodontal ligament cells (hPDLCs) in the presence and absence of osteogenic inducer (L-ascorbic acid, dexamethasone and β-glycerophosphate). The cell growth within the scaffolds in the absence of osteogenic inducers was studied by cell counting kit-8 (CCK-8) assay and scanning electron microscopy (SEM). Alkaline phosphatase (ALP) activity and osteoblastic differentiation markers of hPDLCs in BCP scaffolds were examined in the presence and absence of osteogenic inducers. The cell number of hPDLCs in the BCP scaffolds was less than that of hPDLCs cultured in microplates (control). SEM images showed that cells successfully adhered to the BCP scaffolds and spread amongst the pores; they also produced abundant extracellular cell matrix. In the presence and absence of osteogenic inducers, the ALP activity of hPDLCs within BCP scaffolds was suppressed in varying degrees at all time-points. In the absence of osteogenic inducers, hPDLCs in BCP scaffolds express significant higher levels of osteopontin (OPN) mRNA than the control, and there were no significant differences for Runx2 and osteocalcin (OCN) mRNA levels compared with those cultured in microplates. In the presence of osteogenic inducers, Runx2 expression levels were significantly higher than those in control. OPN and OCN mRNA levels were downregulated slightly. Three-dimensional porous BCP scaffolds are able to stimulate the osteoblastic differentiation of hPDLCs in the presence and absence of osteogenic inducer and may be capable of supporting hPDLC-mediated bone formation.

## Introduction

Periodontitis is a common human infectious disease characterized by loss of connective tissue and bone support (1,2). If left untreated, uncontrolled periodontal tissue destruction eventually leads to tooth migration, increased mobility and tooth loss, impairing chewing function, phonetics and esthetics (3). The ultimate goal of periodontal treatment is to restore the structure and function of the destroyed hard (alveolar bone and cementum) and soft (gingival attachment and periodontal ligament) connective tissues using periodontal-regenerative techniques (4).

Over the last few decades, several therapeutic techniques have been developed for periodontal regeneration, including soft or hard tissue replacement grafts, root surface planning, guided tissue/bone regeneration (GTR/GBR) and the delivery of growth factors or gene therapies (3,5). Recent advances in progenitor cell biology and tissue engineering have enabled the application of cell-based periodontal regeneration using various approaches and principles. Since human periodontal ligament cells (hPDLCs) have multipotential characteristics, the cells are regarded as useful sources for the regeneration of periodontal tissues containing bone, cementum and periodontal ligament (6–8).

Calcium phosphate bioceramics have been used as a bone substitutes to provide a scaffold for cell migration and rapid bone formation because they have unique characteristics compared with other biomaterials. These bioceramics have a close chemical and crystal resemblance to bone mineral and are widely used in orthopedic and dental applications (9). Calcium phosphate scaffolds vary by their physicochemical properties. There are two widely used forms of calcium phosphate ceramics: Hydroxyapatite (HA, Ca_10_[PO_4_]_6_[OH]_2_) and tricalcium phosphate (TCP, Ca_3_[PO_4_]_2_) (9,10). HA scaffolds are strongly osteoconductive but minimally osteogenic, and have been used to repair cranial-facial and other bone defects. However, HA is quite brittle and resorbs slowly when used alone (11). β-tricalcium phosphate (β-TCP) was also utilized in bone regeneration *in vitro* and *in vivo* duo to its osteoconductivity and bioresorbability (11–13). A major drawback of β-TCP scaffolds is that the *in vivo* bone resorption rate of the scaffold exceeds that of the formation of native bone (11). In order to reduce the independent disadvantages of HA and β-TCP, which are largely in opposition to one another, scaffolds composed of these two compounds in various combinations and ratios have been designed.

Biphasic calcium phosphate (BCP) scaffolds consist of variable amounts of HA and β-TCP and their capacity to release calcium and phosphate ions into the local microenvironment may also vary (11). A number of studies have indicated that BCP scaffolds are superior to HA scaffolds and even native bone mineral in mediating bone regeneration (11,14,15). However, the majority of the aforementioned knowledge has been obtained using the organic factors L-ascorbic acid (L-aa), dexamethasone (Dex) and β-glycerophosphate (β-GP), which are osteogenic inducers or osteogenic media *in vitro* (16,17). However, these factors are not expected to be useful *in vivo*. In addition, to our knowledge, few studies have investigated whether BCP exerts intrinsic inductive capacity on the osteoblastic differentiation of human periodontal ligament cells (hPDLCs) in the presence and absence of osteogenic inducers. Thus, the objectives of the present study were to determine the biological properties of BCP scaffolds on hPDLCs, and to evaluate their effect on the osteoblastic differentiation of hPDLCs in the presence and absence of osteogenic inducers.

## Materials and methods

### 

#### Cell culture

The experimental protocol was approved by the Ethics Committee of Sun Yat-Sen University (Guangzhou, China), and informed consent was obtained from all the subjects. The hPDLCs were isolated as previously described (18). In short, fresh periodontal ligament (PDL) tissues were removed from the middle-third of the root and minced, and then were plated in fresh Dulbecco's modified Eagle's medium (DMEM/high glucose, HyClone; GE Healthcare Life Sciences, Logan, UT, USA) containing 20% fetal bovine serum (FBS; Biological Industries, Kibbutz Beit Haemek, Israel) and 2% (v/v) penicillin/streptomycin (Invitrogen Life Technologies, Carlsbad, CA, USA). An explant culture method was used to enable cells to migrate out of the tissue samples in a humidified atmosphere of 5% CO_2_ at 37°C. Once ~80% confluence was met, the cell cultures were collected by trypsinization (0.25% trypsin/EDTA; Invitrogen Life Technologies) and passaged for future studies. Cells at the fifth passage were used in subsequent experiments.

#### Osteoblastic differentiation of hPDLCs (Alizarin red S staining)

The fifth passage of hPDLCs was seeded in a 6-well plate, and cultured in DMEM growth culture medium (GM) supplemented with 10% FBS and 2% penicillin/streptomycin (in the absence of osteogenic inducers) or in osteogenic culture medium (OM) with 10% FBS, 10 mM β-GP, 10^−8^ M Dex, 50 µg/ml L-aa (containing osteogenic inducers). After 7 and 14 days of culture, the process of mineralized matrix deposition formation was observed using an Alizarin red S staining kit (GenMed, Shanghai, China) according to the manufacturer's instructions.

#### Scaffold preparation and cell seeding

BCP scaffolds were supplied by the National Engineering Research Center for Biomaterials (Chengdu, China). The BCP ceramics consist of 70% HA and 30% β-TCP. The porosities of the ceramics were ~70%. Prior to use, all scaffolds were sterilized for 15 min (121°C, 15 bar pressure) by autoclaving. Prior to cell seeding, all scaffolds were soaked in DMEM supplemented with 10% FBS and 2% penicillin/streptomycin at 37°C for 4 h. Then, the hPDLCs were seeded on the scaffolds. The cell-scaffold complexes were maintained in 24-well culture plates and divided into two groups. One of the groups was used for cell proliferation assay and scanning electron microscopy (SEM) observation. The scaffolds were incubated with a suspension of 1×10^5^ cells/ml, using 20 µl cell suspension per scaffold. The cell-scaffold constructs were then cultured in the GM. The other group was used for osteoblastic differentiaton analysis. The scaffolds were incubating with a suspension of 3×10^6^ cells/ml, using 100 µl cell suspension per scaffold. The cell-scaffold complexes were then cultured in the OM. Culture medium was refreshed at 3-day intervals. hPDLCs cultured within plastic culture plates served as the control.

#### Cell proliferation assay

The proliferation rates of hPDLCs within BCP scaffolds were measured in the GM using a Cell Counting kit-8 (CCK-8; Beyotime Institute of Biotechnology, Shanghai, China). In brief, the cells were seeded on scaffolds in 24-well plates at 2×10^3^ cells/per scaffold, with four repeats for each group. Cells were removed from the scaffolds through trypsinization and replaced with 100 µl culture medium on days 1, 2, 3, 4, 5 and 6. The cultures were then transferred to 96-well plates. To each well was added 10 µl CCK-8 solution. The plates were incubated at 37°C for 4 h in a humidified atmosphere of 5% CO_2_. The absorbance of the supernatant was measured with a microplate meter (Infinite 200; Tecan, Männedorf, Switzerland) at 490 nm. Non-seeded scaffolds in the same medium were used as a negative control.

#### SEM analysis

On days 3 and 7 after cell seeding, cell-scaffold complexes were washed with phosphate-buffered saline (PBS) twice and fixed in 2.5% glutaraldehyde for 4 h. Then, the samples were dehydrated in a graded ethanol series and air-dried in tetramethylsilane (Merck Millipore, Darmstadt, Germany). After gold sputtering, the specimens were examined with a Quanta 200 scanning electron microscope (FEI, Hillsboro, OR, USA). As a blank control, BCP scaffolds were investigated, which were incubated in the same culture medium but without cells.

#### Alkaline phosphatase (ALP) activity

After 3 and 7 days of culture, ALP activity was measured by an ALP activity assay kit (Nanjing Jiancheng Biotechnology Co. Ltd., Nanjing, China) following the manufacturer's instructions. All analyses were carried out at three separate preparations of experiments. In brief, the adherent cells were removed from the scaffolds through trypsinization and lysed with PBS, followed by addition of a cell lysis buffer containing 0.1% Triton X-100 to the samples, which were then subjected to three freeze-thaw cycles. Aliquots (50 µl each well) of these supernatants were placed into 24-well plates to which 50 µl ALP substrate solution (2 mM MgCl_2_ and 16 mM *p*-nitrophenyl phosphate) was added. Following incubation at 37°C for 30 min, the reaction was stopped by the addition of 50 µl 0.2 M NaOH, and the liberated *p*-nitrophenol was measured on the Tecan microplate reader at 520 nm.

#### Reverse transcription-quantitative polymerase chain reaction (RT-qPCR)

A total of 3×10^5^ cells were seeded into each scaffold in 24-well plastic culture plates. The expression of Runx2, osteopontin (OPN) and osteocalcin (OCN) mRNA was determined by RT-qPCR on day 21 after initial seeding. The total cell RNA from each scaffold (n=3) was harvested using TRIzol reagent (Invitrogen Life Technologies). Total RNA was used as template for the synthesis of cDNA with Oligo(dT) and RevertAid™ M-MuLV Reverse Transcriptase (MBI/Fermentas, Thermo Fisher Scientific, Pittsburgh, PA, USA). The subsequent PCR amplification reaction was conducted Taq polymerase (Invitrogen Life Technologies) and specific primers. Each PCR was duplicated with the same amount of total RNA. Relative gene expression levels of Runx2, OPN, and OCN were normalized to the expression of the reference gene glyceraldehyde 3-phosphate dehydrogenase (GAPDH). Primers for the selected genes are listed in Table I.

#### Statistical analysis

All experiments were performed three times, with each treatment conducted in triplicate. Means and standard deviations were calculated, and the statistical significance of differences between groups was examined by one-way analysis of variance. SPSS software, version 17.0 (SPSS, Inc., Chicago, IL, USA) was employed for all statistical analysis and differences were considered significant if P<0.05.

## Results

### 

#### Characterization and osteogenic differentiation of hPDLCs

**Figure 1.**
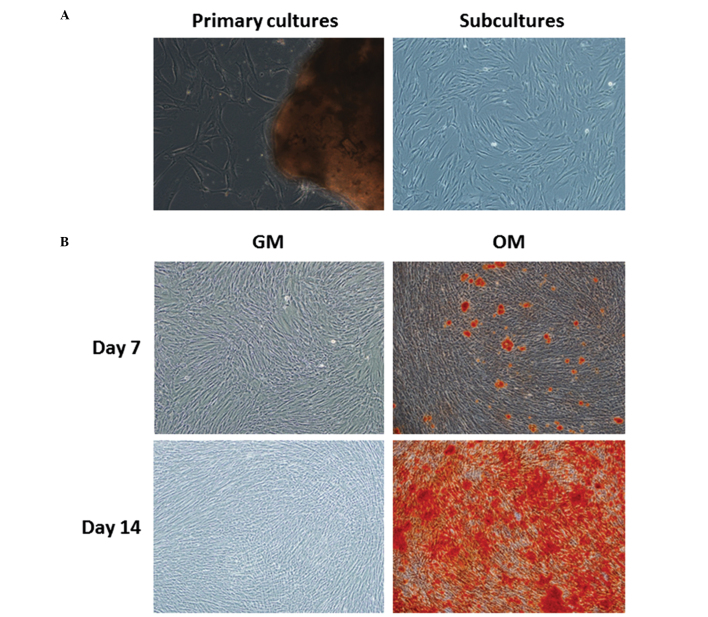
Cell morphology and evaluation of osteogenic differentiation capacity in human periodontal ligament cells (hPDLCs). (A) Primary cultures and subcultures. The isolated cells had typical fibroblastic morphology with triangular, spindle-like and cubic shapes. (B) Evaluation of the osteogenic differentiation of hPDLCs using Alizarin red staining at days 7 and 14. In the absence of osteogenic inducers (growth medium, GM), there wasa no formation of mineralized nodes. In the presence of osteogenic induers (osteogenic medium, OM), hPDLCs were induced into osteoblast-like cells and calcium deposition was detected. Osteogenic culturing of the cells for longer periods of time resulted in more mineralization (magnification, x100).

The isolated hPDLCs had typical fibroblastic morphology with triangular, spindle-like, cuboid and short fusiform shapes (Fig. 1A). The Alizarin red staining results indicated that the differentiation of hPDLCs into osteoblast-like cells and the deposition of calcium occurred only in the presence of β-GP, Dex and L-aa, and that prolonged culturing resulted in the production of larger mineralization nodules (Fig. 1B).

#### Cell proliferation

**Figure 2.**
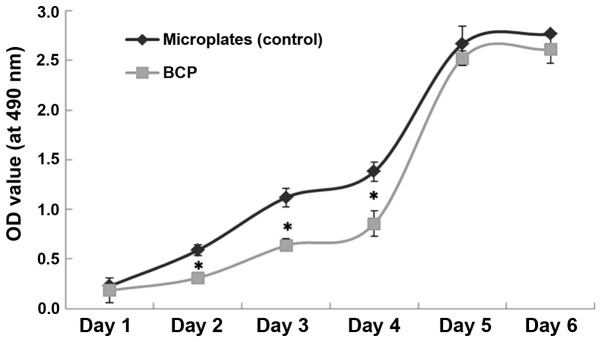
Proliferation of human periodontal ligament cells (hPDLCs) in biphasic calcium phosphate (BCP). The rate of hPDL proliferation in BCP was inhibited significantly compared with control (monolayer cultures in a microplates) at days 2, 3 and 4. However, at days 5 and 6, the proliferation rate of hPDLCs within the BCP was slightly lower than that in the control. *P<0.05 vs. the control. OD, optical density.

The cell number in cell-scaffold complexes from day 1 to day 6 was lower than that in the control group (monolayer cultures in microplates). There was statistically significant difference in cell proliferation levels between the experimental groups and the control at days 2, 3 and 4 (P<0.05). Following 4 days of culture in the GM, however, the cell number within BCP scaffolds was only slightly lower than that in the control (P>0.05; Fig. 2).

#### Observation by SEM

**Figure 3.**
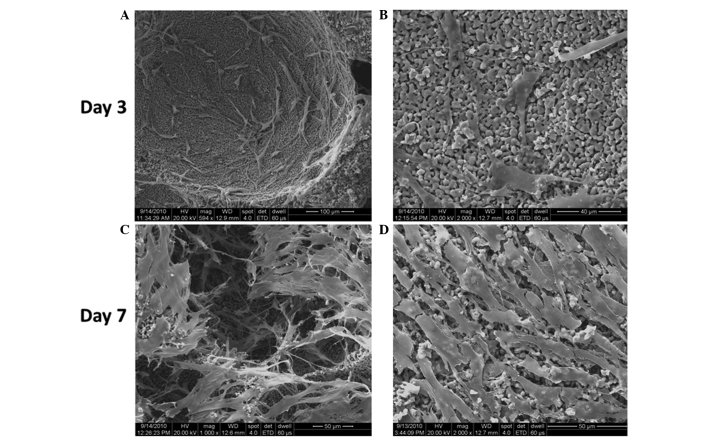
Scanning electron microscopy of the growth of human periodontal ligament cells (hPDLCs) within biphasic calcium phosphate (BCP) scaffolds. (A,B) At day 3, images showed that cells successfully adhered to the BCP scaffolds, were spread over the material surfaces and had migrated inside the pores. The majority of the cells were polygonal in shape. The number of cells adhering on the scaffold surface was relatively low. (C,D) At day 7, large amounts of hPDLCs adhered to the scaffolds and formed multilayered cultures. There were numerous filamentous extracellular matrices on the surface of the cells.

At day 3 after seeding, hPDLCs successfully adhered to the BCP scaffolds, proliferating and spreading among the pores within the three-dimensional network of the scaffold. SEM images demonstrated that the majority of the cells were polygonal in shape, and the number of cells adhering to the scaffold surface was relatively low (Fig. 3A and B). At day 7, large numbers of hPDLCs were observed to have adhered to and proliferated significantly within the BCP scaffolds. There were numerous filamentous extracellular matrices visible on the surface of the cells (Fig. 3C and D).

#### ALP activity

**Figure 4.**
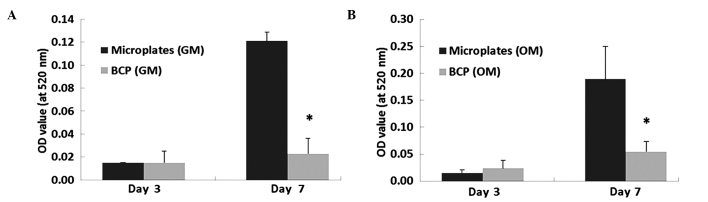
Alkaline phosphatase (ALP) activity of human periodontal ligament cells (hPDLCs) cultured in biphasic calcium phosphate (BCP) scaffolds. (A) In the growth medium (GM), the ALP activity of hPDLCs within BCP scaffolds was comparable with that of the control at day 3; however, it was suppressed confirm suppressed (not promoted) significantly at day 7. (B) In the osteogenic medium (OM), ALP activity showed a similar trend. *P<0.05 vs. the control. OD, optical density.

In the GM and in the OM, the ALP activity gradually increased from day 3 to day 7. The ALP activity of cells cultured on a BCP scaffold exhibited no difference from that of the control cells, cultured in microplates, at day 3 in either medium. However, the ALP activity of hPDLCs within the scaffolds was suppressed significantly compared with the activity of the control hPDLCs at 7 day, and the extent of the suppression differed between in the GM and OM (P<0.05; Fig. 4).

#### BCP scaffolds affect the osteoblastic differentiation of hPDLCs

**Figure 5.**
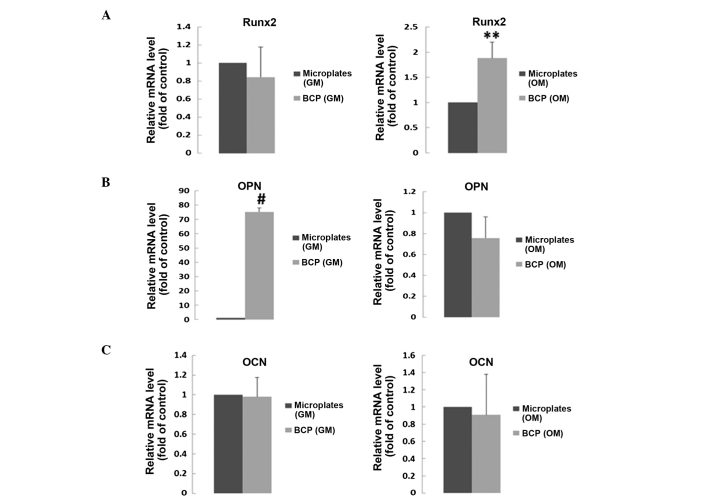
Reverse transcription-quantitative polymerase chain reaction analysis of osteogenic differentiation markers in human periodontal ligament cells (hPDLCs). (A) Runx2, (B) osteopontin (OPN) and (C) osteocalcin (OCN) mRNA. In the growth medium (GM), hPDLCs in biphasic calcium phosphate (BCP) expressed significantly higher levels of OPN compared with the control (monolayer cultures in microplates), and there were no significant differences for Runx2 and OCN mRNA levels between those cultured in BCP and those in microplates. In the osteogenic medium (OM), Runx2 mRNA levels were significantly higher than those in the control, and OPN and OCN mRNA levels were downregulated slightly. *P<0.05, **P<0.01, ^#^P<0.001 vs. the control.

Following 21 days of the induction of osteogenic differentiation, compared with the hPDLCs cultured in plastic culture plates, hPDLCs within BCP scaffolds showed significantly higher levels of Runx2 mRNA in the OM (P<0.01), and OPN in the GM (P<0.001; (Fig. 5A and B). Runx2 mRNA levels in the GM and OPN mRNA levels in the OM were also downregulated, but there was no significant difference in expression level between the experimental group and the control (Fig. 5A and B). OCN mRNA expression levels in the GM and OM were downregulated in the BCP scaffolds but also exhibited no significant changes (P>0.05; Fig. 5C).

## Discussion

Ideal biomedical scaffolds should be biocompatible and nontoxic. One of the first steps in the development of a novel scaffold is the evaluation of its cytotoxicity (3). In the present study, SEM demonstrated that hPDLCs covered most of the outer surfaces of the BCP scaffolds by spreading themselves, and also produced abundant extracellular cell matrix (ECM). ECM plays a very important role in the adhesion, proliferation, and differentiation of cells in the surface of the materials (7,9). Such a response indicates good cytocompatibility and close interaction of the scaffolds with hPDLCs *in vitro*. CCK-8 analysis revealed that the BCP scaffolds were able to inhibit the early but not the late proliferation rate of hPDLCs. This inhibitory effect may be associated with the relatively low number of cell adhering to the scaffold surface initially. Another hypothesis to explain this observation is that in a normal physiological bone-formation process, these progenitor cells proliferate rapidly, and the cells progress down the differentiation pathway at the expense of proliferation (19); that is, cells that are differentiating generally exhibit a lower proliferation rate than those that are not (19,20). The present study showed that BCP significantly induced the expression of OPN mRNA by the hPDLCs in the absence of osteogenic inducers; thus, the cells may have been directed toward osteogenic differentiation causing them to exhibit a low level of proliferation.

ALP activity reflects the early stages of osteogenic differentiation and plays a key role in bone mineralization by initiating and/or promoting the formation of hydroxyapatite crystals in the matrix vesicles of osteoblasts (21). The present study revealed that BCP scaffolds inhibited the ALP activity of hPDLCs. These results conflict with a previous study concerning the behavior of bone cells and dental follicle progenitor cells (22,23). Although the underlying mechanism for this phenomenon is not clear, a hypothesis is that the degradation of BCP scaffolds results in the dissolution and precipitation of calcium and phosphate ions, which competitively inhibits of ALP (18,24,25).

Runx2 is the known to be a key regulator of osteoblast marker genes. Runx2 can also directly stimulate the transcription of osteoblast-related genes such as those encoding OCN and OPN (26). The effect of BCP scaffolds on Runx2 gene expression differed between the GM and OM. In the OM, BCP scaffolds upregulated Runx2 mRNA levels, while in the GM, they did not. This result indicates that BCP scaffolds can promote early osteogenic differentiation in the presence of osteogenic inducer (L-aa, Dex and β-GP). However, this significant upregulation of Runx2 gene expression did not lead to higher levels of OPN and OCN mRNA expression. The lack of a stimulating effect of Runx2 could be explained by the fact that Runx2 triggers the expression of major bone matrix genes during the early stages of osteoblast differentiation, but Runx2 is not essential for the maintenance of the expression of these genes in mature osteoblasts and inhibits osteoblast maturation and mature bone formation (27,28).

It has been reported that OPN is necessary for the initiation of hard tissue mineralization (29). It has been also reported that elevated extracellular levels of inorganic phosphate ions upregulate OPN expression in mouse PDL22 cells in a dose-dependent manner (30). BCP scaffolds may release various concentrations of calcium and inorganic phosphate ions depending on the nature of the local microenvironment (composition, pH, temperature and cell type) (9,24). In the present study, hPDLCs within BCP scaffolds expressed significant higher levels of OPN mRNA than the control in the GM. However, in the OM, the OPN mRNA expression levels were unchanged. In the GM, inorganic phosphate ions released from the degradation of BCP scaffolds could affect OPN gene expression in the cell-scaffold co-cultures, whereas the monolayer (microplate) cultures lack this external source of inorganic phosphate ions. In the OM, the β-GP used to induce mineralization in the osteogenic culture system contains an organic phosphate molecule. Organic phosphate is removed by ALP to release free inorganic phosphate, subsequently providing the chemical potential for the promotion mineral deposition (24). In cell-scaffold co-cultures and monolayer cultures, this released free inorganic phosphate may have an effect. These observations suggested that phosphate ions released from BCP scaffolds may promote OPN gene expression.

BCP is biodegradable and is able to release varying quantities of calcium and phosphate ions into the culture medium or a body fluid (11,12). This release of calcium or phosphate ions has a significant impact on the proliferation and osteoblastic differentiation of hPDLCs (16). The results observed in the present study may be derived from the combined effects of the dissolved ions and the scaffold's architecture and surface chemistry.

In conclusion, the results of present study demonstrate that three-dimensional BCP scaffolds are able to induce the early osteoblastic differentiation of hPDLCs in the presence and absence of osteogenic inducer (L-aa, Dex and β-GP). Therefore, BCP scaffolds may be used as effective templates for guiding the attachment, proliferation and osteoblastic differentiation of hPDLCs *in vitro*.

## Figures and Tables

**Table I.** Primer sequences used for reverse transcription-quantitative polymerase chain reaction.

Genes	Direction	Primer	Size (bp)
Runx2	Forward	CCAACCCACGAATGCACTATC	91
	Reverse	TAGTGAGTGGTGGCGGACATAC
Osteopontin	Forward	GCCGAGGTGATAGTGTGGTT	101
	Reverse	TGAGGTGATGTCCTCGTCTG
Osteocalcin	Forward	CACTCCTCGCCCTATTGGC	148
	Reverse	GCCTGGGTCTCTTCACTACCT
GAPDH	Forward	CATGTTCCAATATGATTCCACC	88
	Reverse	GATGGGATTTCCATTGATGAC	

GAPDH, glyceraldehyde 3-phosphate dehydrogenase.
